# Cross-Reactive Anti-Viral T Cells Increase Prior to an Episode of Viral Reactivation Post Human Lung Transplantation

**DOI:** 10.1371/journal.pone.0056042

**Published:** 2013-02-06

**Authors:** Thi H. O. Nguyen, Glen P. Westall, Tara E. Bull, Aislin C. Meehan, Nicole A. Mifsud, Tom C. Kotsimbos

**Affiliations:** 1 Department of Medicine, Monash University, Central Clinical School, The Alfred Centre, Melbourne, Victoria, Australia; 2 Department of Allergy, Immunology and Respiratory Medicine, The Alfred Hospital, Melbourne, Victoria, Australia; Whitehead Institute, United States of America

## Abstract

Human Cytomegalovirus (CMV) reactivation continues to influence lung transplant outcomes. Cross-reactivity of anti-viral memory T cells against donor human leukocyte antigens (HLA) may be a contributing factor. We identified cross-reactive HLA-A*02:01-restricted CMV-specific cytotoxic T lymphocytes (CTL) co-recognizing the NLVPMVATV (NLV) epitope and HLA-B27. NLV-specific CD8+ T cells were expanded for 13 days from 14 HLA-A*02:01/CMV seropositive healthy donors and 11 lung transplant recipients (LTR) then assessed for the production of IFN-γ and CD107a expression in response to 19 cell lines expressing either single HLA-A or -B class I molecules. In one healthy individual, we observed functional and proliferative cross-reactivity in response to B*27:05 alloantigen, representing approximately 5% of the NLV-specific CTL population. Similar patterns were also observed in one LTR receiving a B27 allograft, revealing that the cross-reactive NLV-specific CTL gradually increased (days 13–193 post-transplant) before a CMV reactivation event (day 270) and reduced to basal levels following viral clearance (day 909). Lung function remained stable with no acute rejection episodes being reported up to 3 years post-transplant. Individualized immunological monitoring of cross-reactive anti-viral T cells will provide further insights into their effects on the allograft and an opportunity to predict sub-clinical CMV reactivation events and immunopathological complications.

## Introduction

Viral infections, in particular human CMV infection, continue to influence clinical outcomes following lung transplantation. Whilst intensive anti-viral prophylactic and pre-emptive strategies following transplantation have reduced the incidence of symptomatic CMV disease in “at-risk” patients, subclinical CMV reactivation in the lung allograft remains associated with poor long term allograft survival [1].

Following a HLA-mismatched lung transplant, alloreactive T cells can infiltrate the lung allograft, resulting in episodes of acute cellular rejection, despite the administration of aggressive immunosuppression. Persistent activities of the same T cells are believed to be the major risk factor for chronic rejection or Bronchiolitis Obliterans Syndrome (BOS) in LTR [2], [3]. There is now clear evidence demonstrating that the total alloreactive T cell repertoire consists of both allo-specific T cells and varying amounts of virus-specific memory T cells [4] that are capable of cross-reactivity towards unrelated HLA alloantigens [5]. In this setting, specific viral infections can potentially heighten immune mechanisms leading to adverse clinical outcomes above and beyond any indirect viral effects.

The capacity of virus-specific memory T cells to cross-react with HLA alloantigens is facilitated by the T cell receptor (TCR), which has been shown to mediate immunological responses in individuals otherwise considered to have been “naïve” to allogeneic stimulation, thereby accounting for the presence of alloreactive memory T cells in individuals with no prior sensitization [6]–[9]. Importantly, cross-reactive anti-viral memory T cells are likely to be less susceptible to immunosuppression regimens and may exponentially expand in the setting of specific viral reactivation. It has been previously proposed that the presence of cross-reactive anti-viral T cells may contribute to a less controllable and easily magnified immunological response that can influence allograft function and survival.

In patients undergoing lung transplantation, we recently described an EBV model of T cell cross-reactivity [10] and explored whether HLA-B*08:01-restricted FLRGRAYGL (FLR)-specific CD8+ T cells cross-recognizing the alloantigen HLA-B*44:02 [11], [12] contributed to allograft dysfunction. Although we demonstrated that cross-reactive FLR-specific CD8+ T cells were detectable and functional in HLA-B8/EBV seropositive LTR that received a HLA-B*44:02 allograft, they did not contribute to allograft dysfunction in the absence of an active EBV infection [10]. Based on this and our previous study showing that low levels of CMV reactivation were sufficient to prime and recruit CMV-specific CD8+ T cells to the lung allograft [13], we suggest that there may be a threshold level of viral reactivation(s) (i.e. magnitude and/or frequency) that is required for cross-reactive virus-specific T cells to become activated and exert deleterious effects on the allograft. Therefore, we now shift our focus towards identifying alloreactive anti-viral T cells in the CMV setting due to its tendency to reactivate much more frequently in our patients compared to EBV.

CMV was a major cause of morbidity and mortality in the early days of lung transplantation when anti-viral prophylaxis was not available. Despite anti-viral prophylaxis however, CMV continues to have a propensity to reactivate post-transplantation in the immunosuppressed host [14], [15], thereby providing a source of ongoing antigenic stimulation. The relatively high frequency of circulating CMV-specific memory T cells [13], [16] and the previously reported cross-reactive nature of T cells towards unrelated HLA alloantigens [4], [17]–[20], produces an immunological environment where increasing viral reactivation may drive recognition of the HLA mismatched allograft. We believe that such a scenario provides further insights to previously reported links between allograft rejection and DNA virus reactivation following transplantation [21]–[23].

The cross-reactive potential of CD8+ T cells specific for the HLA-A*02:01-restricted immunodominant CMV pp65_495–503_ epitope NLVPMVATV (NLV) has been previously reported by independent investigators in healthy individuals, although the specificity of some HLA alloantigens were not completely defined [4], [18], [20]. However, this study showcases a fully characterized novel model of CMV cross-reactivity of NLV-specific CD8+ T cells towards the HLA-B27 molecule (HLA-A-restricted T cells recognizing HLA-B molecules) in both a healthy immunocompetent individual as well as an immunosuppressed LTR. We report for the first time in a clinical setting following lung transplantation that cross-reactive NLV-specific CD8+ T cells remain stable in the setting of persistent alloantigen but significantly increase prior to detectable CMV reactivation. However, in a specific example of CMV reactivation driven increase in cross-reactive anti-viral T cells we did not demonstrate an association between cross-reactive T cells and adverse long term lung allograft outcomes.

## Methods

### Cohort demographics and ethics approval

Eleven HLA-A2 LTR receiving either a HLA-A30, -A31, -A32 or -B27 donor lung allograft between March 2008 and December 2010 (Table 1) and fourteen HLA-A2 healthy individuals (Table 2) were recruited to the study. All LTR received standard triple-therapy immunosuppression and underwent routine surveillance bronchoscopy at approximately 14, 30, 60, 90, 180, 270 and 365 days post-transplant or if clinically indicated [13]. Transbronchial biopsies were assessed for acute cellular rejection and/or CMV pneumonitis according to standard histopathological criteria [24], [25]. Both LTR and healthy controls (HC) provided written consent, with ethics approval granted by The Alfred Hospital (Victoria, Australia) and the Australian Bone Marrow Donor Registry (New South Wales, Australia).

**Table 1:** Patient demographics.

Patient	Age	Gender (D/R)	Primary Disease	CMV status (D/R)	HLA-A, -B (R)	HLA-A, -B (D)
LTR1	62	F/F	COPD	−/−	A2, 24; B27, 44	A3, 30; B18, 65
LTR2	41	M/M	CF	−/−	A2, 3; B7, 62	A1, 32; B8, 27
LTR3	64	F/F	COPD	+/+	A2; B27, 44	A2, 32; B13, 27
LTR4	51	F/M	IPF	−/−	A2, 3; B7, 37	A24, 30; B44, 60
LTR5	35	M/F	IPF	+/+	A2, 3; B7, 18	A11, 33; B27, 58
LTR6	29	F/F	IPF	−/+	A2, 11; B13, 35	A3; B18, 27
LTR7	45	M/M	OB	+/+	A2, 31; B35	A24, 31; B55, 62
LTR8	60	M/M	COPD	+/+	A1, 2; B8, 44	A2, 30; B7, 62
LTR9	64	M/M	COPD	−/+	A2, 24; B13, 40	A2, 31; B40, 57
LTR10	39	M/M	CF	+/−	A1, 2; B7, 27	A11, 31; B51, 55
LTR11	29	F/F	CF-Bronchiectasis	+/−	A2; B39, 44	A2402, 31; B55, 61

Abbreviations: donor (D), recipient (R), female (F), male (M), cystic fibrosis (CF), chronic obstructive pulmonary disease (COPD), idiopathic pulmonary fibrosis (IPF), idiopathic bronchiolitis obliterans (OB).

**Table 2:** Healthy controls (HC) demographics and NLV expansion profiles.

				NLV-specific CD8+ T cells	
HC	HLA-A or -A*	HLA-B or -B*	CMV serology	% *Ex vivo*	% *In vitro*
HC1	2	51, 61	−	NT	0.2
HC2	2, 3	14, 27	−	NT	0.1
HC3	2, 24	07:02, 56:01	+	0.1	23.4
HC4	02:01	15, 44:02	+	1.2	90.5
HC5	02:01, 11	51, 61	+	0.1	69.9
HC6	1, 02:01	07:02, 44:02	+	NT	9.1
HC7	1, 2	08:01, 44:03	+	NT	14
HC8	02:01, 29:02	07:02, 44:03	+	NT	37.6
HC9	2	8, 62	+	NT	97.2
HC10	2	8, 57	+	NT	93.2
HC11	2, 32	35, 44	+	NT	65.95
HC12	2, 3	8, 18	+	2.1	16.3
HC13	2, 3	7, 8	+	1	0.4
HC14	02:01, 03:01	07:02, 45:01	+	1.4	0.2
**Average**				**1.0**	**43.2**
**±SD**				**0.8**	**35.6**
**Range**				**0.1–2.1**	**0.2–97.2**

HLA class I typing, CMV serology status and NLV-specific T cell expansion profiles of healthy individuals. Molecular resolution of HLA class I antigens was available as indicated (4-digit). *In vitro* T cell cultures were derived by autologous stimulation of PBMC with NLV peptide (1 µM) for 13 days in the presence of IL-2 (20 U/ml). Percentages of A2/NLV-tetramer+ T cells were based on the total CD8+ T cell population. HC1 and 2 were excluded from the average, SD and range calculations.

Abbreviation: not tested (NT).

### CMV prophylaxis and monitoring

LTR at risk of CMV reactivation (recipient and/or donor CMV+; R+ and/or D+) received 2 weeks of intravenous ganciclovir treatment (5 mg/kg body density) followed by 5 months of oral valganciclovir anti-viral prophylaxis. In addition, primary D+/R− CMV-mismatch patients received a course of CMV hyperimmune globulin daily in the first month post-transplant. CMV load was measured in bronchoalveolar lavage (BAL) fluid (copies/ml) with the semi-automated COBAS Amplicor CMV monitor test (Roche Diagnostic Systems, NSW, Australia) as described elsewhere [26].

### Lung function

All LTR had routine monitoring of lung function [Spirometry - forced expiratory volume in one second (FEV_1_)] with chronic rejection/BOS defined as a sustained and irreversible loss of FEV_1_ below 80% of Personal Best achieved post-transplant [24], [27].

### Blood samples

Peripheral blood samples from healthy individuals and LTR (taken pre-transplant and at the time of routine bronchoscopy) were collected in heparinized vacutainer tubes. PBMC were isolated by Ficoll-Paque (GE Healthcare, Uppsala, Sweden) density gradient centrifugation and cryopreserved at −180°C until required.

### Cell lines and culture

B-lymphoblastoid cell lines (B-LCL) and HLA class I-transfected cell lines derived from class 1 reduced (C1R) and 721.221 Parental cells (Table 3) were maintained in RPMI (GIBCO, Grand Island, NY), 10% FBS (SAFC Biosciences, Victoria, Australia) and supplements as previously described [28].

**Table 3:** HLA class I typing of cell lines.

Cell Line	HLA-A or -A*	HLA-B or –B*	HLA-C or -C*
9009	01:01	37:01	06:02
9026	26:01	38:01	12:03
9063	32:01	44:02	05:01
9072	31:01	15:01	01:02
T102	2, 29	57, 65	
75083	1, 30	13, 35	6
**C1R Parental** a	**02:01**	**35:03**	**04:01**
C1R.A*01:01	01:01		
C1R.A*02:01	02:01		
C1R.A*03:01	03:01		
C1R.B*08:01		08:01	
C1R.B*18:01		18:01	
C1R.B*27:03		27:03	
C1R.B*27:05		27:05	
C1R.B*27:09		27:09	
C1R.B*35:01		35:01	
C1R.B*35:02		35:02	
C1R.B*35:03		35:03	
C1R.B*44:02		44:02	
C1R.B*44:03		44:03	
C1R.B*57:01		57:01	
721.221 Parental			
721.221.A*29:02	29:02		
721.221.A68	68		
721.221.B53		53	

Molecular resolution of HLA class I antigens was available as indicated (4-digit).

a C1R Parental cell line has no detectable surface expression of HLA-A, low levels of HLA-B35 and normal levels of HLA-Cw4 [51].

### Generation of HLA class I transfectants

Retroviral transduction of C1R.B*27:03, C1R.B*27:05 and C1R.B*27:09 cell lines were carried out as described [29]. Firstly, HLA-B*27:05 cDNA was extracted from the RSV5neoB*27:05 plasmid (kind gift from Dr L. Kjer-Nielsen, The University of Melbourne, Victoria, Australia) using forward and reverse primers: 5′-CCGGAATTCGCCACCATGCGGGTCACGGCGCCCCGAACCCTCC-3′ and 3′-CCGCTCGAGTCAAGCTGTGAGAGACACATCAGAGCCCTGGGCACTGTCG-5′, respectively. HLA-B*27:05 cDNA was cloned into the pGEM-T Easy Vector following manufacturer's instructions (Promega, Madison, WI, USA) before co-transferring into the pMIG vector, (kind gift from Professor D. Vignali, St Jude Children's Research Hospital, Memphis, USA) using EcoR1 and XhoI digestion and ligation techniques to generate pMIG.B*27:05. Site-directed mutagenesis was performed on pMIG.B*27:05 using *PfuTurbo*® DNA polymerase according to manufacturer's instructions (Stratagene, La Jolla, CA, USA) to generate pMIG.B*27:03 (5′ primer: GAGGGGCCGGAGCATTGGGACCGG; 3′ primer: CCGGTCCCAATGCTCCGGCCCCTC) and pMIG.B*27:09 (5′ primer: CGGGTACCACCAGCACGCCTACGACGGC; 3′ primer: GCCGTCGTAGGCGTGCTGGTGGTACCCG) alleles. Retrovirus production was performed via 293T cells for subsequent transduction of C1R Parental cells [29].

### HLA-A2/NLV class I tetramer

R-PE-conjugate tetramer comprising of the HLA-A*02:01/NLVPMVATV (A2/NLV-tetramer) complex was generated as previously described [30]. NLV peptide was synthesized by Genscript (Piscataway, NJ, USA).

### T cell cultures and functional assays

T cell cultures were generated by stimulating PBMC from healthy individuals or LTR with either NLV-pulsed autologous PBMC (irradiated at 3000 Rad) or B-LCL (irradiated at 10,000 Rad) for 13 days (37°C, 5% CO_2_) at a 2∶1 ratio [31]. The functionality of T cell cultures were assessed by (i) proliferation (CFSE assay), (ii) cytokine production (intracellular cytokine staining [ICS] assay) or cytotoxic potential (cell surface expression of degranulation marker CD107a). For proliferation, responder PBMC were stained with CFSE (1 µM, Sigma, St Louis, MO, USA) for exactly 5 minutes at 37°C then washed in the presence of FBS prior to culturing with stimulator cells. Both cytokine production and cytotoxic potential were assessed using a combined CD107a staining and ICS assay [10]. Briefly, PBMC or day 13 T cell cultures (2×10^5^ cells) were stimulated with each cell line (10^5^ cells) for a total of 6 hours in which Brefeldin A (10 µg/ml, Sigma) was added at 2 hours. In CD107a/ICS assays, anti-CD107a FITC (1∶20, clone H4A3, Becton Dickinson [BD], CA, USA) and monensin (3.5 µg/ml, Sigma) were also added at 0 and 1 hour time points, respectively. Stimulation with NLV-pulsed C1R.A*02:01 or NLV peptide (1 µM) were included as positive controls. Negative controls included the background C1R Parental cell line, non-pulsed C1R.A*02:01 or autologous T cells alone. Cells were then labelled with anti-CD8 PE-Cy5 (1∶20, clone HIT8a, BD) and A2/NLV-tetramer (1∶100–200), fixed in 1% paraformaldehyde (ProSciTech, Queensland, Australia) and then permeabilized with 0.3% saponin (Sigma) containing either anti-IFN-γ FITC (1∶50, clone 25723.11, BD) or anti-IFN-γ APC (1∶1000, clone B27, BD) before acquisition using a FACSCalibur (BD). All flow cytometry data was analysed using FlowJo software (Tree Star, Inc., Ashland, OR, USA).

## Results

### Panning for cross-reactive NLV-specific CD8+ T cells towards common HLA molecules

Currently there is a very limited number of human studies characterizing NLV-specific CD8+ T cell cross-reactivity towards unrelated HLA alloantigens [4], [18], which have been shown to demonstrate specificity towards the class I antigens A30, A31, A32 [20]. Considering that following exposure to primary CMV infection there is an establishment of a pool of potentially cross-reactive CMV-specific memory T cells, we sought to define new T cell cross-reactivities towards high frequency HLA alloantigens expressed within the Australian population (Table 3).

**Figure 1:**
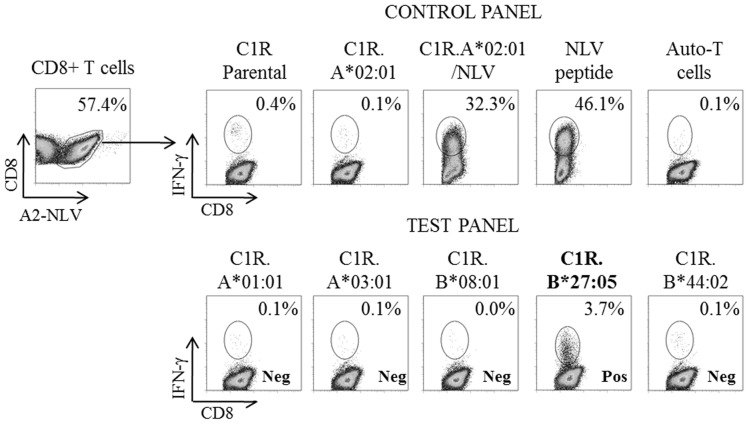
Cross-reactive NLV-specific CD8+ T cells via IFN-γ production. NLV-specific CD8+ T cells from HC5 were expanded for 13 days before performing a 6 hour ICS assay against a panel of transfected cell lines and EBV-LCLs encompassing 6 HLA-A and 13 HLA-B antigens. Cross-reactivity was measured by the production of IFN-γ in response to HLA antigenic stimulation after gating on tetramer+CD8+ T cells. Both the positive controls (C1R.A*02:01/NLV, NLV peptide) and negative controls (C1R Parental, C1R.A*02:01, T cells alone) responded as expected. No cross-reactivity was observed with the test panel, albeit C1R.B*27:05 which had a positive IFN-γ response well above background levels. IFN-γ responses towards 9009, 9026, T102, C1R.B*18:01, C1R.B*35:01, C1R.B*35:02, C1R.B*35:03, C1R.B*44:03, C1R.B*57:01, 721.221 Parental, 721.221.A*29:02, 721.221.A68 and 721.221.B53 were also negative (data not shown).

An expanded pool of day 13 NLV-specific CD8+ T cells from HC3-5 (Table 2) were re-stimulated in a 6 hour ICS assay with a panel of either transfected cell lines or EBV-B-LCL encompassing six HLA-A (A*01:01, A*02:01, A*03:01, A*26:01, A*29:02 and A68) and thirteen HLA-B (B*08:01, B*18:01, B*27:05, B*35:01, B*35:02, B*35:03, B*37:01, B*38:01, B*44:02, B*44:03, B53, B*57:01 and B65). Cross-reactivity was determined by the percentage of NLV-tetramer+ CD8+ T cells producing IFN-γ in response to the cell lines. High levels of IFN-γ production were generated towards the positive controls: NLV-pulsed C1R.A*02:01 (HC3-5; 65.8%, 38.4% and 32.3%, respectively) and NLV peptide alone (HC3-5; 68.8%, 40.7% and 46.1%, respectively). Negative controls were all below 0.1%, except for HC5 with C1R Parental (0.4%) (Figure 1). Background levels of IFN-γ production was only observed in the tetramer-negative CD8+ T cell population in response to B27 alloantigen. High background levels in HC5 are a common occurrence as observed over many independent investigations (data not shown). Of the 19 alloantigens screened, cross-reactivity was only detected towards B*27:05 in HC5, with 3.7% of NLV-specific CD8+ T cells producing IFN-γ upon stimulation with the C1R.B*2705 cell line (Figure 1). As anticipated based on the CMV oligoclonal TCR usage [32], [33], analysis of NLV-specific CD8+ T cells expanded from HC6-11 did not reveal cross-reactivity towards B*27:05 (data not shown).

In addition to panning for new cross-reactive alloantigens recognised by NLV-specific CD8+ T cells, we explored whether HLA-A30, A31 or A32 molecules identified by Morice *et al.* [20] were also present in our cohort of healthy individuals (HC3-11). Day 13 *in vitro* expanded NLV-specific CD8+ T cells were restimulated with three different B-LCL 75083, 9072, 9063 that expressed A30, A31 and A32, respectively (Table 3). However, no evidence of T cell cross-reactivity was observed (data not shown).

Due to the relatively low numbers of *ex vivo* tetramer+ CD8+ T cells in most of our healthy donors (Table 2) and transplant patients, NLV-specific CD8+ T cells were expanded from PBMC in order to increase cell numbers sufficient for the cross-reactivity assays. We have previously compared *ex vivo* versus *in vitro*-expanded cross-reactivity profiles using the public HLA-B*08:01/FLR model and showed that cross-reactivity towards B*44:02 was detected in both conditions, but was more amplified using *in vitro* cultures enabling further functional characterisation [10]. *In vitro* culturing methods have also been used by others to assess cross-reactivity due to the low numbers of tetramer+ CD8+ T cells [4], [20]. Unfortunately for HC5, we were unable to detect any *ex vivo* cross-reactivity (data not shown) due to very low numbers of NLV-tetramer+ CD8+ T cells (≤0.1%).

### Characterising HLA-A*02:01-restricted NLV-specific CD8+ T cell cross-reactivity towards HLA-B*27:05

#### Phenotypic identification

To confirm our previous findings, four independent experiments of HC5 were conducted at different intervals within a one year time period. PBMC were *in vitro* expanded with NLV-pulsed autologous PBMC for 13 days. NLV-specific CD8+ T cells significantly increased in magnitude from very low (below threshold of detection) baseline *ex vivo* frequencies of 0.09±0.06% (range: 0.0–0.1%) to 65.4±16.4% (range: 46.2–80.5%) of the total CD8+ T cell population on day 13. Of these NLV-specific CD8+ T cells cross-reactivity towards B*27:05, measured by IFN-γ production following stimulation with C1R.B*27:05 transfected cell line, was observed with frequencies of 4.9±1.0% (range: 3.7–6.1%) including the initial screening experiment (data not shown).

#### Functional assessment

**Figure 2:**
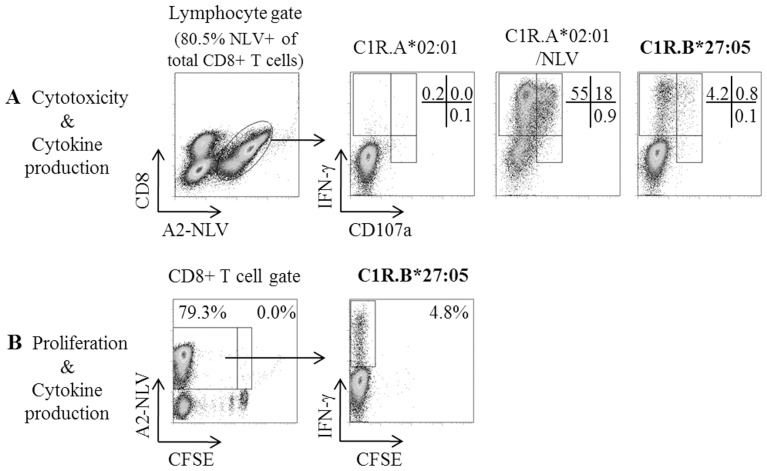
Functional analyses of new cross-reactivity towards B*27:05. Flow cytometric analyses of IFN-γ and CD107a expression were performed after 13 days of NLV-specific T cell expansion from HC5. T cell cultures were stimulated with C1R.A*02:01 (negative control), NLV-pulsed C1R.A*02:01 (positive control) or C1R.B*27:05 (cross-reactive target) for 6 hours in a combined CD107a staining and ICS assay revealing that cross-reactivity was mainly via cytokine production (IFN-γ+) and to a lesser extent dual cytokine/cytotoxic ability (IFN-γ+/CD107a+) (A). Both proliferation and cytokine production were measured in a parallel experiment after CFSE-labelled PBMCs from HC5 were cultured with autologous irradiated NLV-pulsed PBMCs for 13 days before performing a 6 hour ICS assay (B). The lymphocyte gate was based on side scatter versus forward scatter. CD8+ T cells were then gated from lymphocytes using side scatter versus anti-CD8 PE-Cy5.

The day 13 cross-reactive pool of NLV-specific CD8+ T cells were examined for their capacity to (i) induce cytotoxicity, via cell surface expression of degranulation marker CD107a [34], (ii) proliferate, via dilution of CFSE staining [35] and (iii) secrete Th1 cytokine, via production of IFN-γ following stimulation with B*27:05 alloantigen. T cell subsets differentiated by functionality were described as being either single-positive (IFN-γ+ or CD107a+) or double-positive (IFN-γ+CD107a+) in terms of their cytokine production and/or cytotoxic response, respectively. Of the total NLV-specific CD8+ T cell population, responses to C1R.B*27:05 elicited 4.2% IFN-γ+, 0.8% IFN-γ+CD107a+ and 0.1% CD107a+ subset populations, thus suggesting that these cells were dominated by a cytokine producing profile. Both the positive (C1R.A*02:01/NLV; 55.0% IFN-γ+, 18.0% IFN-γ+CD107a+ and 0.9% CD107a+) and negative (C1R.A*02:01; ≤0.2% all subsets) controls generated immune responses as expected (Figure 2A). The entire NLV-specific CD8+ T cell population had proliferated after 13 days as shown by the decreased CFSE expression including the 4.8% cross-reactive CD8+ T cell population following C1R.B*27:05 restimulation (Figure 2B). Collectively, this data demonstrated both confirmation and functionality of our model of HLA-A*02:01-restricted NLV-specific CD8+ T cell cross-reactivity in response to HLA-B*27:05.

### HLA-B27 allelic variation influences the magnitude of CD8+ T cell cross-reactivity

**Figure 3:**
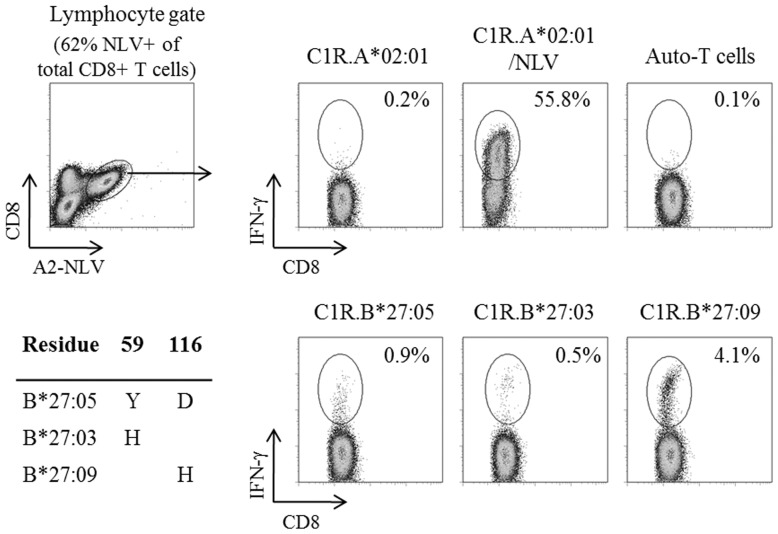
Influence of cross-reactivity by B27 allelic subtypes. Site-directed mutagenesis of the pMIG.B*27:05 vector was performed to generate B*27:03- and B*27:09-specific retroviruses for transducing C1R Parental cells. Comparison of cross-reactive NLV-specific IFN-γ responses between B*27:05, B*27:03 and B*27:09 was then carried out following a 6 hour stimulation and ICS assay of day 13 NLV-specific CD8+ T cells (HC5) with the B27-specific cell lines as well as positive (C1R.A*02:01/NLV) and negative controls (C1R.A*02:01, Auto-T cells).

In a well-characterized model of EBV TCR cross-reactivity, HLA-B*08:01-restricted FLR-specific CD8+ T cells were able to recognise the B*44:02 alloantigen, but not B*44:03 [11], [12]. These two B44 allelic subtypes differ by a single amino acid substitution, aspartate (D) for B*44:02 or leucine (L) for B*44:03, at position 156 of the alpha 2 domain, which contributes to disparate alloreactive profiles [28] as well as impacting on clinical transplant outcomes [36], [37]. To determine whether the A*02:01/NLV cross-reactivity model was influenced by B27 allelic variation, day 13 *in vitro* expanded NLV-specific CD8+ T cells generated from HC5 were restimulated with C1R transfectants expressing either B*27:03, B*27:05 or B*27:09 in a 6 hour ICS assay (Table 3). Using B*27:05 as the consensus sequence, B*27:03 and B*27:09 alleles differ by a single amino acid substitution at position 59 (tyrosine (Y) to histidine (H)) and position 116 (D to H), respectively (Figure 3). Site-directed mutagenesis of B*27:05 cDNA was used to generate the C1R transfectants for B*27:03 and B*27:09 subtypes. Comparison of immune reactivity of NLV-specific CD8+ T cells directed against each of the B27 alleles demonstrated that the B*27:09 allele (4.1%) generated the strongest frequency of IFN-γ production, followed by B*27:05 (0.9%) and then B*27:03 (0.5%) (Figure 3). The same cross-reactive T cell immune hierarchy of B*27:09>B*27:05>B*27:03 was observed in three independent experiments (data not shown).

### Measuring the cross-reactive T cell potential in HLA mismatched lung allografts

To determine the potential impact of NLV-specific CD8+ T cell cross-reactivity on the specific clinical allograft outcomes of lung function and acute rejection, as well as CMV primary infection or reactivation and survival following transplantation, 11 HLA-A*02:01 LTR who received either an HLA-A30 (LTR1, 4, 8), A31 (LTR7, 9–11), A32 (LTR2, 3) or B27 (LTR2, 3, 5, 6) bilateral lung allograft (Table 1) were investigated for (i) the presence and expansion of NLV-specific CD8+ T cells and (ii) their ability to recognise HLA alloantigens based on our newly identified model of A*02:01/NLV T cell cross-reactivity (B27) and the previously published models (A30, A31, A32) [20].

**Figure 4:**
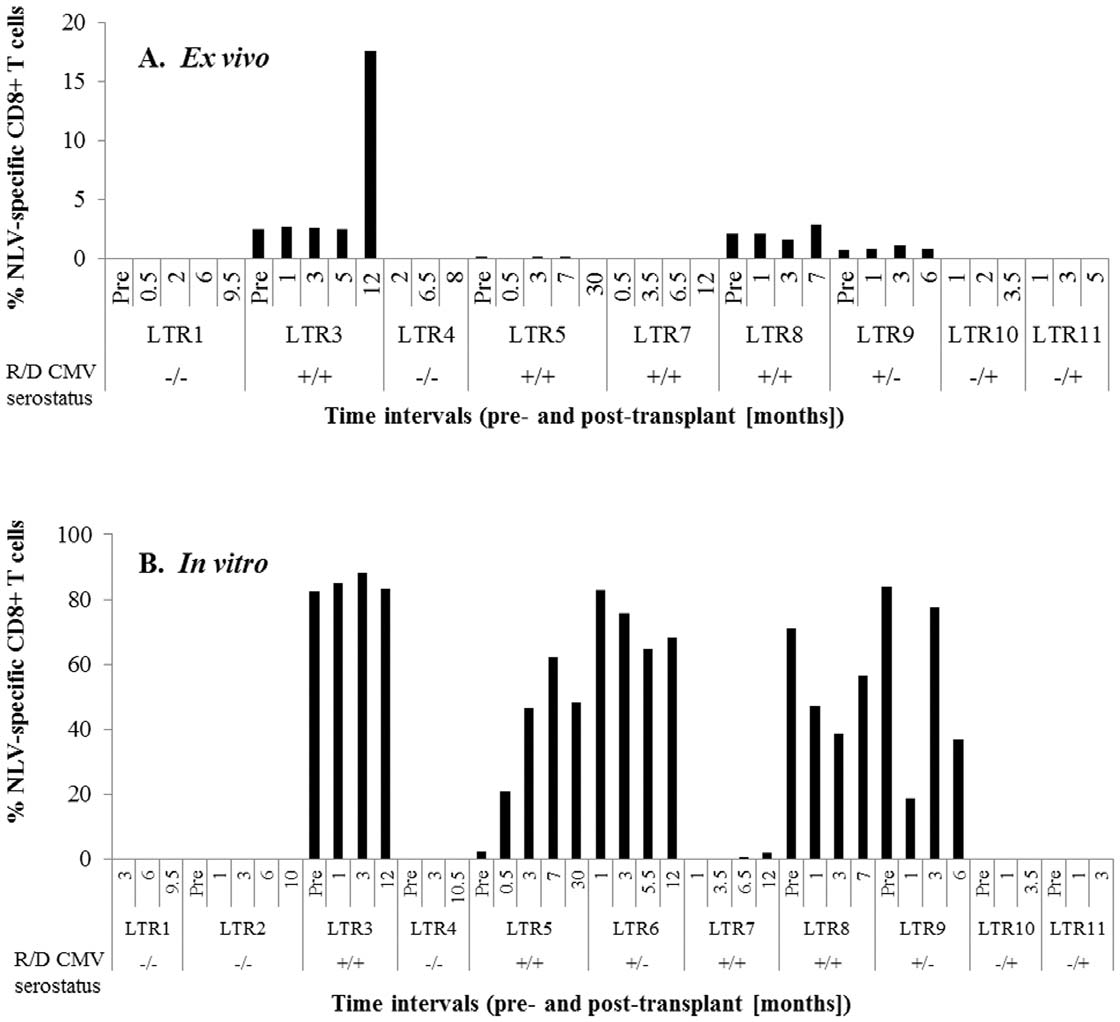
Expansion profiles of NLV-specific CD8+ T cells in LTR. NLV-specific CD8+ T cell frequencies were measured on day 0 (A) and after 13 days of NLV peptide stimulation (B) based on the total CD8+ T cell population, where available. CMV serostatus of the recipients and donors are indicated in the graphs.

Firstly, levels of circulating NLV-specific CD8+ T cells were measured from pre-transplant up to 12 months post-transplant, where available. *Ex vivo* analysis of A*02:01/NLV-tetramer+ cells revealed undetectable levels (<0.1%) in one CMV seropositive (+) LTR (LTR7) and as expected in four CMV seronegative (−) LTR (LTR1, 4, 10, 11). However, a range of 0.1–17.6% was detected in four CMV+ LTR (LTR3, 5, 8, 9) (Figure 4A). LTR2 (CMV−) and LTR6 (CMV+) were not evaluated. Following antigen-specific expansion of the memory T cell pool, the presence of NLV-specific CD8+ T cells was detected in all six CMV+ LTR (range 0.1–88.1%; LTR3, 5–9) (Figure 4B). Although LTR10 and LTR11 received CMV+ allografts, we did not detect any NLV-specific CD8+ T cells within the first 3.5 months post-transplant.

**Figure 5:**
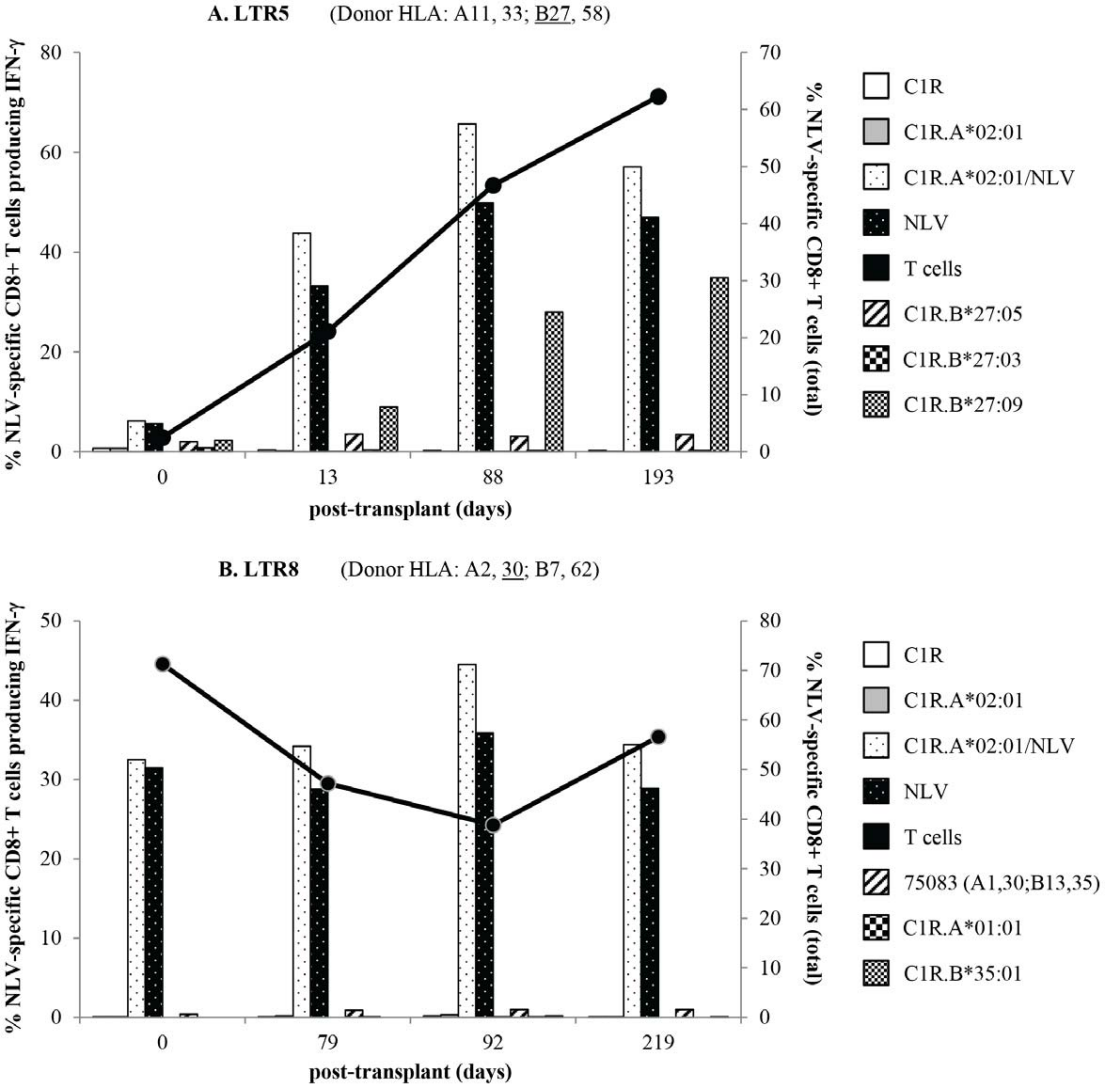
Longitudinal analysis of LTR cross-reactivity. NLV-specific cross-reactivity responses against B27 and A30 were measured against time after lung transplant in LTR5 ([A] top) and LTR8 ([B] bottom), respectively. Following a 6 hour ICS assay of day 13 T cell cultures, B*27:09 cross-reactivity significantly increased over time in LTR5, whereas A30 cross-reactivity remained stable post-transplant in LTR8. Percentages were based on IFN-γ production of NLV-specific +CD8+ T cells. The secondary axis represents the percentage of NLV-specific CD8+ T cells gated on total CD8+ T cells (circles).

Secondly, quantitation of NLV-specific CD8+ T cell cross-reactivity toward the HLA alloantigens A30, A31, A32 or B27 was measured (IFN-γ production) following *in vitro* stimulation of the expanded pool of NLV-specific CD8+ T cells with B-LCL expressing the respective mismatched HLA molecules. Of the 11 LTR, T cell cross-reactivity was only detected in LTR5 and LTR8, who had received a B27 and an A30 lung allograft, respectively (Figure 5).

In LTR5, *ex vivo* levels of NLV-specific CD8+ T cells ranged between 0.0–0.1% from pre-transplant to 193 days post-transplant (Figure 4A). However, peptide-induced *in vitro* expansion of NLV-specific CD8+ T cells yielded frequencies of 2.5% pre-transplant to 62.3% at 193 days post-transplant (Figure 5A, total NLV-specific CD8+ T cells). More importantly, cross-reactivity of NLV-specific CD8+ T cells towards two of the three B27 alleles tested was observed, with B*27:09 cross-reactivity gradually increasing from 2.3% to 34.9% of NLV-specific CD8+ T cells producing IFN-γ from pre-transplant to 193 days post-transplant. Although, cross-reactivity towards B*27:05 was evident the level remained relatively stable over time (Figure 5A). This data suggested that the B*27:09 allele was the most favourable cross-reactive alloantigen.

For LTR8, *ex vivo* levels of 1.6–2.9% of NLV-specific CD8+ T cells (data not shown) expanded upon antigenic stimulation to 38.8–71.3% from pre-transplant to 219 days post-transplant (Figure 5B, total NLV-specific CD8+ T cells). Based on a report of NLV-specific CD8+ T cell cross-reactivity towards A30 alloantigen [20] we showed a minimal IFN-γ response of 0.4–1.0% that remained stable throughout the pre-transplant to 219 days post-transplant time period (Figure 5B). No NLV-specific CD8+ T cell cross-reactivity was observed towards A31 or A32 alloantigens in this group of LTR.

### Increase in cross-reactive T cells is dependent on availability of an antigen source

**Figure 6:**
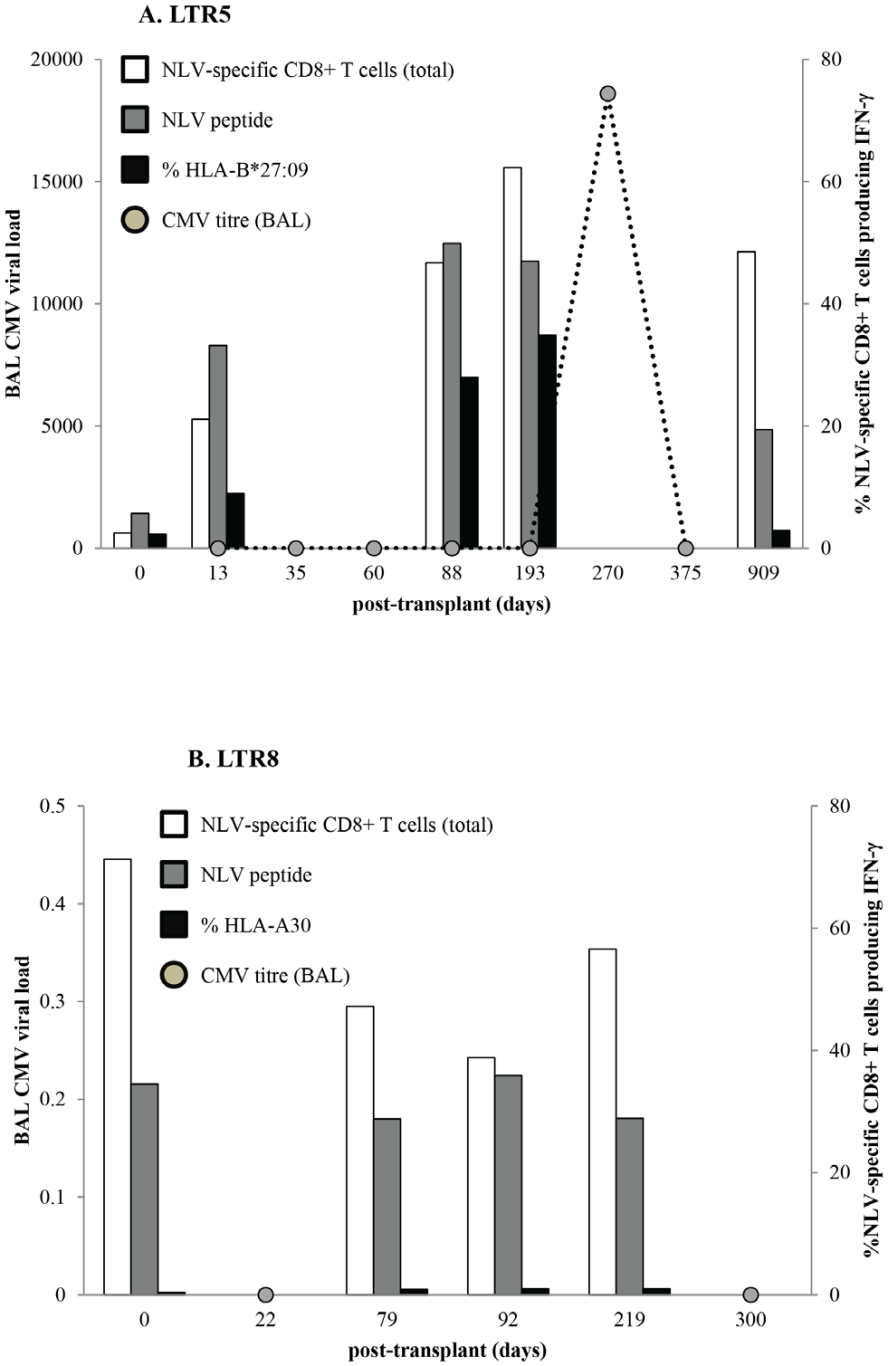
Presence of CMV reactivation increases magnitude of cross-reactivity against the allograft. Comparison of CMV viral load in the BAL (grey circles, left y-axis) and NLV-specific cross-reactivity responses towards B27 and A30 (6 hour ICS assay using day 13 T cells) were measured against time after lung transplant in LTR5 (top) and LTR8 (bottom), respectively. A CMV reactivation episode was classified for viral titres above 10,000. LTR5 experienced CMV reactivation at day 270 post-transplant but had ceased after day 375. A steady increase in B*27:09-cross-reactivity response based on %IFN-γ production of tetramer+CD8+ gated cells (black bars, right y-axis) and %tetramer+CD8+ cells of total CD8+ T cells (white bars, right y-axis) were observed prior to CMV reactivation event at day 193 but had dropped to pre-transplant levels after CMV reactivation had ceased. For LTR8, BAL CMV viral titre was not detected post-transplant. In alignment, A30 cross-reactivity and %tetramer+CD8+ cells of total CD8+ T cells remained consistent post-transplant.

Of the 11 LTR, 2 (18%) were at risk of experiencing primary CMV disease despite anti-viral prophylaxis (R−/D+: LTR10, 11), 6 (55%) were at risk of CMV reactivation (R+/D− or R+/D+; LTR3, 5–9), whilst 3 (27%) were of no risk (R−/D−; LTR1, 2, 4) and hence did not receive CMV prophylaxis (Table 1). In all LTR, except LTR5, there was no primary CMV infection or reactivation event within the first 12 months post-transplant based on CMV viral load monitoring, although LTR10 and LTR11 were only assessed to 3 months post-transplant due to sample availability. For LTR5, CMV reactivation was detected on day 270 post-transplant by a positive PCR for CMV DNA and a viral titre of 18,600 copies/ml. Interestingly, CMV pneumonitis was also evident on day 270 in the transbronchial biopsy sample. However, negative PCR results and undetectable viral loads were recorded both prior to (days 13–193) and following (day 375) the CMV reactivation episode (Figure 6A). Although routine CMV prophylaxis had ceased on day 159 (5 months post-transplant), LTR5 received an additional 2 weeks of intravenous ganciclovir treatment from day 270 of CMV reactivation (9 months), followed by oral valganciclovir treatment (450 mg: morning and evening) up until 24 months post-transplant.

Although CMV viremia in LTR5 was undetectable in BAL samples on days 13, 35, 60, 88 and 193 post-transplantation, we quantitatively observed an increase in both conventional and cross-reactive, towards HLA-B*27:09, NLV-specific CD8+ T cells (Figure 6A). A clinically relevant active CMV reactivation was measured on day 270 post-transplantation and following CMV treatment intervention strategies and clearance of the virus, only the cross-reactive NLV-specific CD8+ T cell pool returned to baseline frequencies (day 909) (Figure 6A). Conversely, in the absence of CMV viremia there was no increase in cross-reactive NLV-specific CD8+ T cells as demonstrated by LTR8 (Figure 6B).

### Clinical dynamics of lung function, acute cellular rejection and pulmonary associated survival were not influenced by presence of cross-reactive T cells

Of the 11 LTR, NLV-specific CD8+ T cell cross-reactivity was detected only in LTR5 (B*27:09 and B*27:05) and LTR8 (A30). Our *in vitro* studies demonstrated that the presence of either exogenous peptide (LTR5, 8) or CMV viremia (LTR5) significantly magnified the frequency of these cross-reactive T cells, which raises the possibility that these cells can contribute to adverse clinical events against the lung allograft expressing the target HLA alloantigen. To determine the impact of NLV-specific CD8+ T cell cross-reactivity on allograft function we monitored longitudinal measurements of lung function, acute cellular rejection and survival on the background of potent immunosuppression.

There was no association between the potential cross-reactive T cell dynamics and physiological lung function (as measured by % Personal Best) in either LTR5 and LTR8, who both exhibited excellent lung function (>90%) up to 24 months post-transplant (data not shown). Transbronchial biopsy samples were evaluated for the presence of acute cellular rejection within the first 12 months post-transplant. There was no evidence of cellular infiltrate or change in tissue architecture reported for either LTR5 or LTR8. Indeed, all LTR were alive at 24 months following transplantation.

Collectively these data show that cross-reactive NLV-specific CD8+ T cells did not necessarily contribute to any clinical manifestations of allograft dysfunction in the single CMV reactivation scenario that could be examined. Whilst we observed a significant increase in cross-reactive T cells frequency following CMV reactivation, we suspect that a single viral reactivation event (particularly if it is low level and relatively short lived) may not be sufficient to drive cross-reactivity associated destructive lung immunopathology in adequately immunosuppressed LTR.

## Discussion

Since the first human report of a cross-reactive EBV-specific CD8+ T cell recognizing the unrelated HLA alloantigen B*4402 [11] was described, there have been an array of publications identifying human cross-reactive virus-specific T cells directed towards both class I and class II HLA alloantigens in CMV [4], [17]–[20], EBV [4], [11], [12], [38], HSV-2 [39], Influenza [4] and Varicella zoster virus (VZV) [4] models. However, these reports examined T cell cross-reactivity in healthy individuals and could only speculate about the implications of this mechanism in contributing to destructive immunopathology associated with either allograft rejection or graft versus host disease (GvHD) in a transplant setting.

Only recently have there been two human reports in a real clinical setting examining the contribution of cross-reactive virus-specific T cells towards either allograft dysfunction in LTR (from our group) [10] or GvHD in hematopoietic stem cell transplant recipients [40]. Whilst both studies demonstrated the presence of cross-reactive virus-specific T cells and their contribution to the alloreactive T cell pool in patient cohorts, we and Melenhorst *et al.* were unable to associate cross-reactive virus-specific T cells with either episodes of allograft dysfunction and rejection or incidences of GvHD, respectively. In our study [10], we specifically measured EBV reactivation in order to better frame our T cells cross-reactivity results. However, as there was no active EBV infection in our patient cohort we concluded that the presence of cross-reactive T cells was not enough to mediating allograft rejection alone and that the frequency and extent of active viral infection was likely to be important. Hence, we have since focussed on a CMV study in the setting of either primary infection (CMV mismatch) or reactivation, which occurs much more frequently (7.2% or 17.5%, respectively from 2006–2008; n = 97) and constitutes severe morbidity and mortality associated complications following lung transplantation. In this setting, we proposed that viral antigenic stimulation *in vivo* has the potential to magnify cross-reactive CMV-specific T cell responses towards specifically targeted HLA alloantigen(s), thereby contributing to a destructive immunopathology and promoting allograft rejection or loss. In support, we have previously reported that CMV-specific memory T cells can account for up to 20% of the total circulating T cells early post-transplantation and uniformly increases following episodes of significant CMV reactivation [13], [41].

For the first time, we describe the cross-reactivity of HLA-A*02:01-restricted CMV-specific CD8+ T cell towards the common HLA-B27 antigen. Functional characterisation of NLV-specific CD8+ T cell cross-reactivity was determined by both IFN-γ production and cell surface expression of CD107a. Our model utilizes the predominance of HLA-A*02:01 in our general population (over 40%) and our lung transplant cohort (37%) as well as the immunodominant nature of the NLV epitope [42], [43]. Cross-reactivity between HLA-A and -B groups is a new paradigm for CTL recognition with only one other example reported recently whereby HLA-A*02:01-restricted VZV-specific CTLs cross-reacted with HLA-B*55:01 cell lines in one VZV-seropositive healthy individual [4] and one VZV-seronegative kidney transplant patient following VZV vaccination [44]. The fact that we observed similar HLA-A to -B cross-reactivity in both a healthy donor and a LTR suggests that this mechanism of T cell recognition may be more common than previously considered.

We assessed the clinical interrelationships between the presence and dynamics of cross-reactive CD8+ T cells against clinical outcomes including lung function, acute cellular rejection and pulmonary associated survival following lung transplantation. As only LTR5 had increasing cross-reactive NLV-specific CD8+ T cells in the setting of a CMV reactivation profile, but stable lung allograft function at 24 months we were limited in making any sweeping conclusions regarding increased anti-viral cross reactivity and poorer lung allograft outcomes. Importantly, the proportion of cross-reactive CD8+ T cells in relation to the total NLV-specific CD8+ T cell population in LTR5 declined back to pre-transplant levels once the virus was cleared. This poses an important question; should these cross-reactive T cells continue to dominate in the setting of multiple hits of CMV reactivation, then would this accelerate allograft loss? To address this critical question, future studies measuring cross-reactive T cells in cohorts experiencing multiple CMV-related events would be useful, as well as the expansion of known cross-reactivities between CMV and HLA molecules.

The presence of stable circulating cross-reactive CD8+ T cells seen in LTR8 with no CMV reactivation may suggest that there may be compartmental issues in terms of using peripheral blood for our assays rather than BAL mononuclear cells that reside within the lung allograft, however there are technical difficulties associated with obtaining sufficient cell numbers for these investigations. In support, we have previously reported that CMV-specific CD8+ T cell dynamics in both the blood and the lung allograft reflect viral reactivation following lung transplantation [13]. Another consideration is that our experiments involved primarily *in vitro* cultures to enhance the signal from very low starting numbers of *ex vivo* virus-specific (tetramer+) T cells. Our previous study in the EBV model compared *ex vivo* and *in vitro* FLR-specific cross-reactive responses towards B*44:02 and although they were functionally different (cytotoxic versus cytolytic, respectively), the strength and hierarchy of the response was highly comparable [10].

Moving from the classic T cell cross-reactivity model of the EBV gamma herpesvirus to a more evolved CMV beta herpesvirus raises a very different immunological scenario for consideration. The EBV-specific TCR repertoire that recognises the HLA-B*08:01-restricted FLR peptide is widely public and predominantly consists of either the cross-reactive LC13 clone, expressed by almost all individuals who do not co-express HLA-B*44:02/05, or the non-cross-reactive CF34 clone [11], [12], [45]. Whereas, the CMV-specific TCR repertoire for the HLA-A2-restricted NLV epitope is much more diverse [43], [46], [47] and may be an important mechanism to counteract CMV's highly evolved immune evasion strategies. Although public NLV-specific TCRs have been described [47], [48], the oligoclonal nature helps explain why we and others have identified the relatively private specificity of NLV-specific T cell cross-reactivity between individuals of either undefined HLA alloantigens [4], [18] or A30, A31, A32 [20] and B27 (this study). Yet, the allopeptide(s) presented by these HLA molecules remain unknown. By chance, we were still able to detect comparative B27 T cell cross-reactivity in a healthy donor and a lung transplant patient as well as confirm A30 directed T cell cross-reactivity as reported by Morice *et al.* [20] in one patient using a limited sample size, suggesting that these cross-reactive T cells may not be so “private”. This now leads to question whether we would find more commonality between NLV-specific T cell cross-reactivity profiles as we increasing our cohort numbers or whether these cross-reactive TCR's are truly private and only unique to a few individuals. Further studies are currently being investigated to determine whether HC5 and LTR5 share a common TCR profile.

The cross-reactivity hierarchy between the B27 subtypes may be explained by the location of the variant residues. For example, B*27:05 and B*27:09 differ by a single substitution at residue 116 (D to H, respectively) which is located on the floor of the peptide binding groove. Structural studies by Fiorillo and colleagues show CD8+ T cell functional disparities between B*27:05 and B*27:09 in their engagement of self and viral peptides [49]. However, a full understanding of the mechanistic basis of our B27-cross-reactivity model with structural studies will require the discovery of the allopeptide.

We observed one LTR example of a single episodic increase of cross-reactive NLV-specific CD8+ T cells was not associated with adverse clinical lung immunopathology and allograft deterioration. Interestingly, these cross-reactive T cells significantly increased in frequency prior to the onset of a clinically significant CMV reactivation and were then shown to decrease back to baseline levels following CMV viremia clearance. In contrast, significant increases of the cross-reactive T cell pool were not seen in the setting of persistent alloantigen exposure under current immunosuppression strategies. The findings of this study suggest that at the very least a threshold level of anti-viral cross-reactivity may be required to mediate allograft rejection. Whilst the findings of this study does not directly support recent experimental evidence in a murine model examining T cell cross-reactivity, where Lymphocytic Choriomeningitis Virus-specific T cells were shown to mediate skin allograft rejection [50], it should be remembered that these murine studies were performed in a highly artificial and controlled environment, without the administration of immunosuppression.

Our previous studies showed the ability of cross-reactive EBV-specific T cells to induce immune reactivity towards HLA molecules expressed on both PBMC and BAL mononuclear cells [10], [28], [31]. However, we were unable to demonstrate the clinical impact of these T cells in mediating allograft dysfunction [10]. In addition to the requirement for active viral infection, cross-reactive T cells may exert tissue-specific recognition of HLA alloantigens. A seminal study by D'Orsogna *et al.* [44] confirmed that recognition of allogeneic HLA molecules by virus-specific memory T cells is dependent on self-peptide presentation by the allogeneic target cell. The more readily testable EBV model yielded key observations with EBNA3A-specific T cells showing weak recognition of HLA-B*44:02 target cells due to lack of EEYLQAFTY peptide presentation in specific tissues. Further studies examining the ability of differential lung allograft tissue (epithelial versus endothelial) to (i) naturally process and present HLA/self-peptide complexes and (ii) decipher their ability to be specifically targeted by cross-reactive anti-viral T cells are currently being explored.

In conclusion, we have demonstrated that cross-reactive NLV-specific CD8+ T cells remain stable in the setting of persistent alloantigen but significantly increase prior to a clinically relevant CMV reactivation event. We speculate that a series of immunological events may be required to align in order for cross-reactive virus-specific T cells to exert physiological damage on transplanted allograft, especially in the setting of potent immunosuppressive regimens. These events include (i) an active viral infection as a sustainable antigen source, (ii) the magnitude (high titres) and frequency (more than one episode) of viremia requiring clinical intervention, (iii) the significant increase in frequency of cross-reactive T cells and (iv) the tissue-specific expression of the targeted HLA/peptide complex. Finally, we speculate that discordance between persistently increased cross-reactive anti-viral T cells and CMV viremia that has subsided may be the first sign that these anti-viral T cells are influencing allograft immunopathology.
